# Trial of remote continuous versus intermittent NEWS monitoring after major surgery (TRaCINg): a feasibility randomised controlled trial

**DOI:** 10.1186/s40814-020-00709-8

**Published:** 2020-11-23

**Authors:** C. L. Downey, J. Croft, G. Ainsworth, H. Buckley, B. Shinkins, R. Randell, J. M. Brown, D. G. Jayne

**Affiliations:** 1 Leeds Institute of Medical Research at St James’s, University of Leeds, Leeds, LS9 7TF UK; 2 St James’s University Hospital, Level 7, Clinical Sciences Building, Leeds, LS9 7TF UK; 3 Clinical Trials Research Unit, Leeds Institute of Clinical Trials Research, University of Leeds, Leeds, LS2 9NL UK; 4 Academic Unit of Health Economics, University of Leeds, Leeds, LS2 9NL UK; 5 School of Healthcare, Baines Wing, University of Leeds, Leeds, LS2 9JT UK

**Keywords:** Continuous, Early warning score, Feasibility, Vital signs, Monitoring, Surgery, Complications

## Abstract

**Background:**

Despite medical advances, major surgery remains high risk with up to 44% of patients experiencing postoperative complications. Early recognition of postoperative complications is crucial in reducing morbidity and preventing long-term disability. The current standard of care is intermittent manual vital signs monitoring, but new wearable remote monitors offer the benefits of continuous vital signs monitoring without limiting the patient’s mobility. The aim of this study was to evaluate the feasibility, acceptability and clinical outcomes of continuous remote monitoring after major surgery.

**Methods:**

The study was a randomised, controlled, unblinded, parallel group, feasibility trial. Adult patients undergoing elective major surgery were randomly assigned to receive continuous remote monitoring and normal National Early Warning Score (NEWS) monitoring (intervention group) or normal NEWS monitoring alone (control group). Continuous remote monitoring was achieved using the SensiumVitals® wireless patch which is worn on the patient’s chest and monitors heart rate, respiratory rate and temperature continuously, and alerts the nurse when there is deviation from pre-set physiological norms. Feasibility was assessed by evaluating recruitment rate, adherence to protocol and randomisation and the amount of missing data. Clinical outcomes included time to antibiotics in cases of sepsis, length of hospital stay, number of critical care admissions and rate of hospital readmission within 30 days of discharge.

**Results:**

One hundred and thirty-six patients were randomised between October 2018 and April 2019: 67 to the control group and 69 to the intervention group. Recruitment was completed prior to the 12 month target with a high rate of eligibility and consent. Missing data was limited only to questionnaire responses; no participants were lost to follow-up and only one participant was withdrawn due to loss of capacity. The number of patients classed as ‘drop-out’ due to design (8.1%) were less than anticipated, and there were no participants who crossed over into the alternative trial allocation group. Seventeen participants in the intervention group (28%) did not adhere to the monitoring protocol. No formal comparisons between arms was undertaken; however, participants had fewer unplanned critical care admissions (1 versus 5) and had a shorter average length of hospital stay (11.6 days (95% confidence interval 9.5–13.7 days) versus 16.2 days (95% confidence interval 11.3–21.2 days)) in the continuous vital signs monitoring group. The time taken to receive antibiotics in cases of sepsis was similar in both arms. A cost-utility analysis indicated that the remote monitoring system was cost-saving when compared to standard NEWS monitoring alone.

**Conclusions:**

It is feasible to perform a large-scale randomised controlled trial of continuous remote monitoring after major surgery. Progression to a definitive multicentre randomised controlled trial would be appropriate, taking consideration of factors, such as patient adherence, that might mask the potential benefit of additional monitoring.

**Trial registration:**

ISRCTN registry with study ID ISRCTN16601772. Registered 30 August 2017.

**Supplementary Information:**

**Supplementary information** accompanies this paper at 10.1186/s40814-020-00709-8.

## Key messages regarding feasibility

- What uncertainties existed regarding the feasibility?
  A wearable wireless continuous monitoring system was implemented on an elective general surgical ward, and compared to traditional intermittent vital signs monitoring. Prior to the design of a multicentre randomised controlled trial to assess the effectiveness of continuous monitoring on patient outcomes, we aimed to assess the feasibility of the study protocol on recruitment, estimated recruitment rate, levels of adherence to protocol, estimated amount of missing data and potential optimal outcome measures.
- What are the key feasibility findings?
  The findings of this feasibility study suggest that progression to a definitive multicentre randomised controlled trial would be appropriate. There were high rates of patient recruitment. There were fewer than anticipated ‘drop-outs’ and no participants crossed over into the alternative trial allocation group. Missing data was also limited; no participants were lost to follow-up and only one participant was withdrawn. Patient acceptability was excellent, although patient adherence to protocol could be improved.
- What are the implications of the feasibility findings for the design of the main study?
  Participants should be individually randomised and stratified to minimise the baseline differences between the two treatment arms; ASA might be replaced by more specific risk stratification tools. The intra-cluster correlation co-efficient should be taken into account in the sample size calculation to account for potential clustering of outcomes at bay or ward level. Care should also be taken to monitor and address inadequacies in other areas that might mask the potential benefit of additional monitoring, such as patient adherence.

## Background

Patients having major surgery are at high risk of complications, some of which can be life-threatening. Rates of complications can be as high as 33–44% in patients undergoing surgery for gastro-intestinal cancers [1]. Patients who develop postoperative complications become progressively unwell, often over a short period of time. Early recognition of postoperative complications is crucial in reducing morbidity and preventing long-term disability; for patients with septic shock, there is an 8% increase in mortality for every hour of delay in antibiotic administration [2]. Early detection and treatment of complications minimises the need for Level II/III care and produces significant cost savings [3].

The recording of patients’ vital signs is a key aspect of monitoring for complications. In England, the National Early Warning Score (NEWS) is the standard of care recommended by the Royal College of Physicians [4]. Vital sign scores are summated to provide a numerical score that gives an indication of the patient’s physiological status. Typically, in the postoperative period, NEWS will be calculated half hourly for the first few hours; if the patient remains stable, the frequency will decrease to 2-hourly and then 4-hourly, until the patient is ready for discharge when the NEWS may be recorded only twice a day.

Although NEWS has proven benefit, it suffers from several drawbacks [5]. NEWS relies on manual observations, is time-consuming and open to user interpretation. Vital signs are taken at predetermined intervals with patient deterioration possible between recordings. It has been suggested that the gap between observations is one of the primary failings of the NEWS system [6].

A solution to the problem of inadequate monitoring frequency is continuous monitoring at the bedside. Until recently, continuous monitoring has been limited to Level II/III care due to its cost and limitations to patient mobilisation [7]. Recent advances in wearable technology have heralded the advent of remote wireless monitors. It is hypothesised that the remote continuous monitoring system, as an adjunct to standard vital signs monitoring, will allow earlier detection of patient deterioration. This should reduce morbidity, which in turn should result in a decreased need for Level II/III care.

Demonstrating significant benefit over intermittent monitoring to offset the practical and economic implications of continuous monitoring requires large, well-controlled studies in high-risk populations to demonstrate significant differences in clinical outcomes, such as critical care admissions. Given the complexity of the intervention, before a definitive trial is designed, there is the need for a feasibility study focussed on not only clinical outcome measures but also patient acceptability and adherence. In addition, a feasibility study will allow the identification of barriers to recruitment, estimate protocol adherence and allow optimisation of the technology and trial design.

The main aim of the study was to determine the feasibility of performing a large-scale individually randomised controlled trial of continuous remote monitoring after major surgery. Secondary aims were to informally assess the potential safety, potential efficacy, acceptability and potential cost utility of a wearable, remote monitoring system for patients after major surgery, as compared to standard monitoring with the NEWS system alone.

## Methods

### Ethical approval and consent to participate

Ethical approval was granted on 10^th^ October 2017 by the Yorkshire & The Humber–Leeds West Research Ethics Committee, ref: 17/YH/0180. Informed written consent to participate was obtained from all participants in the study.

### Trial design

This was a single-centre, feasibility, randomised, controlled, parallel group trial of continuous remote vital signs monitoring for patients undergoing major elective general surgery. Participants were individually randomised on a 1:1 basis to receive either remote monitoring plus NEWS or monitoring by NEWS alone. The planned recruitment period was 12 months. Participant follow-up extended to 30 days after hospital discharge.

The study was registered on the ISRCTN registry (ISRCTN 16601772). The study protocol has been published separately [8]. No changes were made to the registered protocol. The trial was performed in accordance with Good Clinical Practice guidelines and the Declaration of Helsinki, and is presented according to the CONSORT statement principles [9] and the CONSORT 2010 extension to randomised pilot and feasibility trials [10].

### Study setting

All participants were recruited on the admissions ward at St James’s University Hospital, Leeds, United Kingdom.

### Eligibility criteria

Patients were selected on the basis that they were undergoing elective major abdominal surgery. Patients were identified, recruited and consented for inclusion in the trial on the day of their surgery by a research nurse or a clinical fellow.

#### Inclusion criteria

- Patients undergoing elective abdominal surgery
- Patients with the capacity to provide informed, written consent on admission
- All ages ≥ 18 years

#### Exclusion criteria

- Patients undergoing emergency surgery
- Allergy to adhesives on electrodes
- Cardiac pacemaker in situ

### Randomisation

Following confirmation of eligibility and written informed consent, participants were randomised into the trial by a research nurse or clinical fellow. Randomisation was performed centrally using the University of Leeds Clinical Trials Research Unit (CTRU) 24-h randomisation service, either via the telephone or the CTRU website.

Participants were randomised on a 1:1 basis and allocated a unique trial number. Randomisation was conducted using permuted stratified block randomisation with variable block size with gender (male/female) and ASA grade (grades 1–4) as stratification factors.

The randomisation sequence was generated by a statistician in the Leeds Clinical Trials Research Unit and computer-generated using SAS 9.4 (SAS Institute Inc., United States of America, 2013). This sequence was implemented and delivered by programmers through the Leeds CTRU Gen24 system, a dedicated telephone and web-based randomisation service.

### Interventions

Patients randomised to the intervention arm received a SensiumVitals® remote monitoring patch and standard NEWS monitoring. The SensiumVitals® patch monitors heart rate, respiratory rate and temperature continuously. The data are transmitted wirelessly every 2 min to a mobile device carried by the nurse that alerts when there is deviation from pre-set physiological norms. Although there are a number of similar devices on the market, the SensiumVitals® system was chosen for evaluation in this work as it is CE-marked and the company was in a position to support a timely evaluation before aiming for widespread adoption.

The patch was applied as soon as possible after the patient came out of theatre. Participants admitted to Level II/III care after surgery had the patch activated once they returned to a participating Level I ward.

Two surgical wards participated in the study: male and female. The male ward housed 25 beds, whilst the female ward housed 28 beds. The patch was activated on arrival to the ward and the patient’s nurse carried a mobile device to alert them if the vital signs strayed outside of normal parameters. Remote monitoring data were also accessible on the ward computer screens for wider access. There was no dedicated telemetry screen for the patch data. Nursing staff were provided with thorough training before the commencement of the study. If the mobile devices alerted the nursing staff to abnormal vital signs, the ensuing clinical response was not mandated, but left to the nurse’s discretion within the boundaries of hospital protocols.

Patients in the control arm received standard NEWS monitoring alone. All usual nursing and medical care were permitted within both arms of the trial.

The patients were to remain in their allocated study arm for the duration of their hospital stay. If a remotely monitored patient was moved to a critical care bed during their admission, the remote monitoring was temporarily suspended pending reinstatement if they returned to a participating ward. Every effort was made to ensure that participants remained in the study arm to which they were originally allocated, and any non-adherence was recorded.

### Blinding

Blinding was not applicable for this study. Neither the patients nor the nursing staff could be blinded to the intervention received. The data collection was performed by a research nurse and clinical fellow, who were both administering the monitoring device, and so were necessarily unblinded. However, the objective methods of collecting the outcome data minimised the risk of bias. These data were taken from the clinical records made by the patients’ usual care teams, including a succession of junior medical staff on rotation, who were unaware of the study. In addition, the predefined criteria for the outcome measures provided minimal scope for interpretation of their presence or absence by the data collection team. The clinical fellow performed the analysis alongside an unblinded statistician.

### Primary outcome measures

There were no changes to the outcome measures pre-specified in the published protocol [8]. Outcome measures were assessed after the trial had closed. No interim analyses were performed. The primary outcome measures were:

- Recruitment rate
- Information on the ideal method of randomisation, which will include calculation of intra-cluster correlation co-efficient to investigate whether there is any inherent clustering in outcomes based on which ward bay a participant is admitted to.
- Adherence to protocol, and reasons for non-adherence, as defined by the number of patients who do not receive the correct type of monitoring as per randomisation (and reasons for this) and the number of patients who do not wear the patch for their entire hospital stay or at least 5 days during their admission.
- Amount of missing clinical data and loss-to-follow-up.
- Optimal outcome measures to test efficacy as assessed by the amount of missing data and the summary statistics for each potential outcome.
- Estimation of parameters to input into the sample size calculation for a definitive trial.

### Secondary outcome measures

The secondary outcome measures were:

- Number of postoperative complications, defined and scored according to the Clavien-Dindo classification of surgical complications [11].
- Number of reinterventions, defined and scored according to the Clavien-Dindo classification of surgical complications.
- Time to antibiotics in cases of sepsis, as defined by the presence of a likely source of infection and 2 or more of the following criteria: temperature > 38.3 °C or < 36.0 °C, tachycardia > 90 beats per minute, tachypnoea > 20 breaths per minute, pCO^2^ < 4.3 kPa, hyperglycaemia (blood glucose > 6.6 mmol/L) in the absence of diabetes mellitus, acutely altered mental status, white blood cell count > 12 × 10^9^/L or < 4 × 10^9^/L [12].
- Number of HDU/ICU admissions.
- Length of stay in HDU/ICU.
- Total length of stay in hospital.
- 30-day hospital readmission rate.
- Patient acceptability assessed using a questionnaire and by calculating the number of patients not wearing a patch for at least 5 days.
- The comparative cost-effectiveness of the SensiumVitals® remote monitoring system versus standard NEWS monitoring from an NHS payer perspective.

### Sample size

As the trial was designed to assess the feasibility of conducting a future definitive large-scale trial, a formal power calculation was not considered appropriate as efficacy was not being formally evaluated.

The simulation work of Teare et al. [13] recommends that 60 participants per group is sufficient to estimate event rates in feasibility trials with binary endpoints. This work provides the justification of the sample size of at least 120 subjects in a two-armed study. It was anticipated that a binary endpoint would be the outcome measure of interest; for continuous endpoints, however, a smaller sample of 70 participants (35 per group) would have been sufficient.

It was anticipated that 20% of participants would not be admitted to a participating ward; these participants were classed as ‘drop-out’ due to design, and were not included in the modified intention-to-treat analysis.

### Planned analyses

Analysis was carried out following the principles of modified intention-to-treat (ITT). The modified ITT (mITT) population included all participants randomised to the trial, analysed according to the treatment group to which they were randomised, regardless of adherence to the protocol. The modified ITT (mITT) population excluded any participants who were classed as ‘drop-out’ due to design (i.e. those who were never admitted to a participating ward).

Baseline characteristics were summarised descriptively overall and by trial arm. Quantitative secondary outcome measures were summarised descriptively using appropriate summary statistics both overall and by trial arm (mean, standard deviation, range and median for continuous outcomes and frequency and percentages for categorical measures). Proportions of missing data are also presented. Missing data does not refer to missing sensor data, only to missing clinical data as collected on the Case Report Forms.

As this is a feasibility study, no formal comparison between the study arms was undertaken. Summaries were produced by subgroup to determine any differences between low- and high-risk patients. High-risk patients were defined as ASA > 2 undergoing Major+ surgery, or ASA ≥ 2 with a perioperative critical care admission.

Analysis was carried out in SPSS (Version 23.0 or later, IBM Corp., New York, USA), apart from the ICC calculations and the sample size calculations. The ICC was estimated from a one-way analysis of variance (ANOVA) in Stata (Release 17, StataCorp, Texas, USA) using a cluster size of 7. This cluster size was chosen as it represents the mean number of patients in each bay (range 4–8).

An estimation of the sample size for a definitive trial was calculated using a two-group Satterthwaite *t* test of equal means and unequal variances using NQuery (Version 3.0, Statistical Solutions Ltd., Massachusetts, USA).

A cost-utility analysis was conducted using decision-analytic modelling. The base-case analysis took an NHS perspective and the time horizon for the model was 6 weeks post-surgery. The model is provided in Additional file 1. Transition probabilities for the model were taken exclusively from the study data. A targeted literature search was conducted to identify relevant studies that reported data for the model parameters for costs and utility values (details available on request from the corresponding author). Outcomes comparing the SensiumVitals® remote monitoring system with standard NEWS monitoring were presented as incremental cost-effectiveness ratios (ICERs). To characterise uncertainty in the model parameters, a probabilistic sensitivity analysis was carried out, based on ten thousand simulations parameterizing the model from the pre-specified parameter distributions. The simulated ICERs were presented in a cost-effectiveness plane. All analyses were conducted in Microsoft Excel (Microsoft Corporation, Redmond, WA, USA). The cost-effectiveness plane was produced in R (Version 3.4.1, R Foundation for Statistical Computing, Massachusetts, USA).

### Progression criteria

The pre-specified criteria to indicate that progression to a definitive randomised controlled trial would be feasible were:

- The recruitment of 120 patients within 12 months who received monitoring on the trial
- Missing data limited to no more than 20% attrition (drop-out by design, loss to follow-up or withdrawal from monitoring)

## Results

**Fig. 1:**
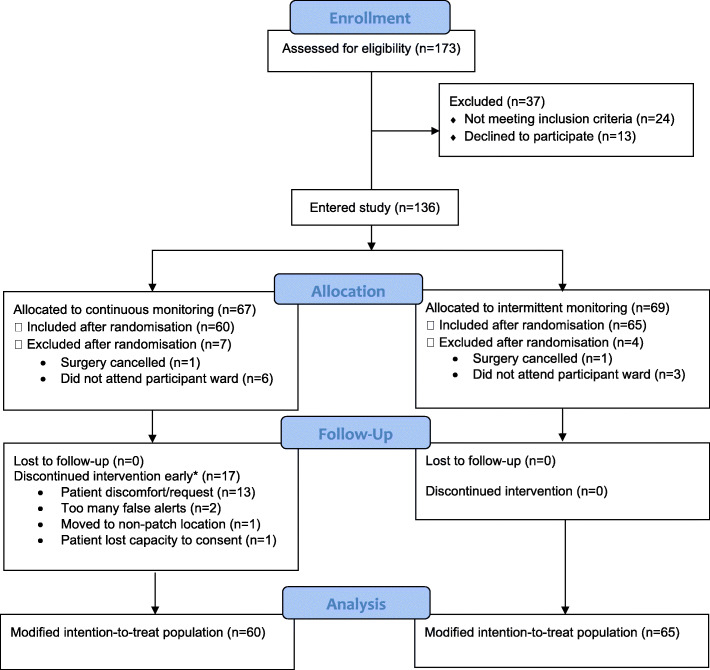
CONSORT flow diagram for the trial (*Early discontinuation of the intervention was defined as not wearing a patch for at least 5 days or until discharge from hospital)

One hundred and thirty-six patients were included in the study between October 2017 and April 2018. Follow-up ended in May 2018. A patient flow chart is presented in Fig. 1, and patient characteristics in Table 1.

**Table 1:** Baseline patient characteristics and intraoperative demographics

	NEWS alone *n* = 65	SensiumVitals® + NEWS *n* = 60	Total *n* = 125
Males *n* (%)	35 (28.0%)	31 (24.8%)	66 (52.5%)
Females *n* (%)	30 (24.0%)	29 (23.2%)	59 (47.2%)
Age (mean, range)	62 (22 – 87)	65 (36–85)	63 (22–87)
ASA *n* (%)			
1	6 (4.8%)	4 (3.2%)	10 (8.0%)
2	40 (32.0%)	39 (31.2%)	79 (63.2%)
3	19 (15.2%)	17 (13.6%)	36 (28.8%)
4	0	0	0
Not documented	0	0	0
Type of surgery			
Colonic resection	55 (44.0%)	53 (42.4%)	108 (86.4%)
Small bowel resection	1 (0.8%)	3 (2.4%)	4 (3.2%)
Other*	9 (7.2%)	4 (3.2%)	13 (10.4%)
Mode of surgery for colonic resections			
Laparoscopic	32 (25.6%)	32 (25.6%)	64 (51.2%)
Open	18 (14.4%)	17 (13.6%)	35 (28.0%)
Converted	3 (2.4%)	2 (1.6%)	5 (4.0%)
Assisted	2 (1.6%)	2 (1.6%)	4 (3.2%)

*Further details on the type of surgery undergone by the participants is presented in Additional file 2

Of the 173 patients assessed for eligibility, 24 were excluded prior to approach because they did not meet the inclusion criteria: ten patients went to theatre before approach; seven patients had a pacemaker; four patients were in source isolation; two patients lacked capacity; one patient was in a conflicting research trial. Of the 149 patients approached, 13 declined to participate because they did not want to be involved in research in general or because they felt too anxious about their impending surgeries.

Consent was obtained from 136 patients, all of whom were subsequently randomised. Sixty-seven patients were allocated to receive continuous monitoring alongside standard care. Sixty-nine patients were allocated to the control group.

Eleven patients were excluded from the modified intention-to-treat population. These were patients who had their surgery cancelled on the day (*n* = 2: one participant per arm) or who did not return to a participating ward (*n* = 9: six in the intervention arm, three in the control arm). The modified intention-to-treat population consisted of 60 participants in the intervention arm, and 65 participants in the control arm. One participant was withdrawn from the study when they lost capacity to consent in the post-operative period and did not regain capacity; this participant was in the intervention arm.

The participant characteristics at baseline were well balanced across both arms of the study (Table 1). Both arms were similar in terms of the types of surgery the participants underwent. Further details on the type of surgery undergone by the participants are presented in Additional file 2.

### Primary outcome measures

#### Recruitment

The eligibility rate of all considered patients was 86.1% (13.9% of patients were ineligible). The recruitment rate was 91.3% out of those eligible. The proportion of patients who were classed as ‘drop-out’ by design was 8.1%. The trial recruited to target and ahead of time.

#### Assessment of randomisation

The intra-cluster correlation co-efficient was calculated based on 121 observations; participants who were transferred between different bays during their admission (*n* = 3) were excluded from this analysis, as was the participant who was withdrawn from the study (*n* = 1). Length of hospital stay was chosen as the outcome measure of interest for this analysis for three reasons:

1. It is an outcome likely to be of relevance in a definitive study
2. There were likely to be data for every participant in the trial
3. Other potential candidates for the primary outcome in a definitive study were likely to exhibit too few events in the feasibility trial to calculate a meaningful ICC. This includes outcome measures such as readmission rates, time to antibiotics in sepsis and critical care admissions

The ICC was found to be moderate (ICC = 0.06150, 95% CI = 0, 0.18839), indicating that the bay into which a participant was admitted had some effect on their length of hospital stay.

#### Adherence to protocol

Participants in the control arm were 100% adherent to protocol. From the intervention arm, 17 participants (28.3%) did not adhere to protocol. Eight participants (13.3%) did not receive the intervention at any point during their admission as they declined the patch after their return to the ward. Of note, 7 of the 8 participants who did not have the patch applied were categorised as high-risk. Nine participants discontinued the intervention before discharge and within 5 days of application. Reasons for this included patient discomfort or skin reaction (*n* = 5), too many false alerts (*n* = 2), transfer to non-participating ward (*n* = 1) and incorrect assumption of imminent discharge (*n* = 1).

#### Amount of missing clinical data and loss-to-follow-up

No participants were lost to follow-up. One participant was withdrawn from the study after they lost capacity to consent during their hospital admission; no further study data was collected after the participant lost capacity. Data up to the point of lost capacity has been included in the analysis. Missing data was limited to questionnaire responses; 14 patients did not fill in the questionnaire as they left hospital before it could be administered.

#### Optimal outcome measures to test efficacy

Assessment of the optimal outcome measures took into consideration the amount of missing data and summary statistics for each potential outcome. Two potential outcome measures displayed compelling differences between the two trial arms: rate of critical care admission and length of hospital stay. There was no missing data for any of these outcome measures; however, data from the one withdrawn participant was censored at the time of withdrawal, forfeiting data regarding their length of hospital stay and any subsequent complications.

#### Estimation of parameters to input into the sample size calculation for a definitive RCT

Estimation of the sample size range for a definitive trial was calculated using the observed effect sizes for length of hospital stay.

Based on the mean length of hospital stay from the feasibility study data and using the point estimate for the ICC, the target sample size for a definitive trial was calculated as 602 participants (301 per arm), which provides 80% power at the 5% significance level to detect a 2-day difference in length of hospital stay, allowing for 15% attrition. To provide 90% power, 808 participants would be required (404 per arm). Further sample size calculations using the limits of the confidence intervals for the ICC and the chosen endpoint are provided as Additional file 3.

### Secondary outcome measures

#### Postoperative complications and reinterventions

There were more complications in the control arm than the intervention arm in every Clavien-Dindo classification grou p[11], as summarised in Table 2. This was especially evident in the number of participants experiencing major complications (Clavien-Dindo III or IV): 10 in the control arm (15.5%) versus 3 in the intervention arm (5.0%).

**Table 2:** Summary of postoperative complications and reinterventions

	NEWS alone *n* = 65	SensiumVitals® + NEWS *n* = 60	Total *n* = 125
Number of complications (all)	180	124	304
I	85	59	144
II	82	62	144
IIIa	3	1	4
IIIb	5	1	6
IVa	3	0	3
IVb	2	1	3
Number of participants experiencing major complications (Clavien-Dindo > II)	13 (10.4%)	4 (3.2%)	17 (13.6%)
Number of reinterventions			
Medical	170	121	291
Radiological	3	1	4
Surgical	7	2	9
Total	180	124	304

The proportion of participants receiving at least one re-intervention, as defined by the Clavien-Dindo complications classification, was 76.9% in the control arm and 80.0% in the intervention group.

#### Time to antibiotics in cases of sepsis

From the modified intention-to-treat population, 35 participants were suspected of having sepsis at least once during their hospital admission: 16 from the control arm (24.6%) and 19 from the intervention arm (31.7%). Of these, sepsis was confirmed in 22 cases. Twenty-one patients received antibiotics: 9 from the control arm (75%) and 12 from the intervention arm (52.5%). The sources of sepsis are provided as Additional file 4.

The mean time to antibiotics was 527 min in the control arm (range 56–1474 min, 95% CI 199 min, 856 min) and 551 min in the intervention arm (range 14–1165 min, 95% CI 296 min, 805 min).

In the intervention arm, 5 out of 19 events were first identified by the SensiumVitals® remote monitoring system (26.3%). The remaining events were first identified by the NEWS system.

#### HDU/ICU admissions

Six participants were admitted to HDU or ICU from a participating ward following surgery. Five participants were from the control arm with an average critical care stay of 3 days. One participant was from the intervention arm; their length of stay in critical care is unknown as they were withdrawn from the study due to lack of capacity (see Table 3).

**Table 3:** Summary of critical care admissions, length of hospital stay, readmission rates and deaths

	NEWS alone *n* = 65	SensiumVitals® + NEWS *n* = 60
Level II/III admissions		
*n*	5	1
Length of stay in Level II/III (days)		
Mean (s.d.)	3 (2.0)	(.)^a^
Length of stay in hospital (days)		
Mean (s.d.)	16.2 (20.3)	11.6 (8.2)
95% confidence interval	11.3–21.2	9.5–13.7
Readmissions^b^		
*n* (%)	5 (7.7%)	6 (10.2%)
95% confidence interval	2.5– 17.0%	3.8– 12.8%
Inpatient deaths	0	1

^a^The length of stay in Level II/III is unknown for this participant as they were withdrawn due to loss of capacity

^b^The denominator for readmissions was the total number of participants in each respective arm of the study; however, readmission data was not collected for the participant who was withdrawn from the study

#### Total length of stay in hospital

As shown in Table 3, participants in the control arm had a longer average length of hospital stay (16.2 days, 95% CI 11.3 days, 21.2 days) compared to those in the intervention arm (11.6 days. 95% CI 9.5 days, 13.7 days).

#### Thirty-day readmission rate

Eleven participants (8.9%) were readmitted to hospital within 30 days of discharge from their index admission. Five were from the control arm (7.7%, 95% CI 2.5%, 17.0%) and six from the intervention arm (10.2%, 95% CI 3.8%, 20.8%). All readmissions were emergency admissions.

### Subgroup analysis of secondary outcome measures

In the pre-specified subgroup analysis, the intervention arm had a slightly higher proportion of high-risk participants (48.3%) than the control arm (41.5%) (Table 4).

**Table 4:** Summary of secondary outcomes by risk subgroup

	NEWS alone: high-risk	SensiumVitals® + NEWS: high-risk	NEWS alone: low-risk	SensiumVitals® + NEWS: low-risk
*n* (%)	27 (41.5%)	29 (48.3%)	38 (58.5%)	31 (51.7%)
Number of complications (all)	91	70	89	54
Number of major complications (Clavien-Dindo > 2)	8	3	5	0
Sepsis events *n* (%)	8 (29.6%)	12 (41.4%)	8 (21.1%)	7 (22.6%)
Time to antibiotics in cases of sepsis (minutes)				
*n*	6	5	6	4
Mean	588	433	466	697
95% confidence interval	0–1247	54– 813	281– 651	381– 1013
HDU/ICU admissions				
*n*	4	1	1	0
Length of stay in HDU/ICU (days)				
Mean	3.5	(.)^a^	1	N/A
Length of stay in hospital (days)				
*n*	27	28	38	31
Mean	23.3	15.7	11.2	7.9
95% confidence interval	12.4–34.2	11.9–19.5	8.8–13.7	7.1–8.6
Readmissions				
*n* (%)	4 (14.8%)	4 (14.3%)	1 (2.6%)	2 (6.5%)
95% confidence interval	4.2–33.7%	4.0–32.7%	0.1–13.8%	0.8–21.4%

^a^The length of stay in Level II/III is unknown for this participant as they were withdrawn due to loss of capacity

Of the high-risk participants, those in the control arm experienced more unplanned critical care admissions, but fewer sepsis events than those in the intervention arm. High-risk participants in the control arm had a longer average length of hospital stay (23.3 days, 95% CI 12.4, 34.2 days) compared to those in the intervention group (15.7 days, 95% CI 11.9, 19.5 days), but were no more likely to be readmitted back to hospital within 30 days of discharge.

Subgroup analysis of low-risk participants revealed similar trends across all secondary outcome measures: lower rates of major complications, sepsis events and critical care admissions in the intervention group. Low-risk participants in the control arm had a longer average length of hospital stay (11.2 days, 95% CI 8.8, 13.7 days) compared to those in the intervention group (7.9 days, 95% CI 7.1, 8.6 days), but were less likely to be readmitted back to hospital within 30 days of discharge. These findings are summarised in Table 4.

### Patient acceptability

**Fig. 2:**
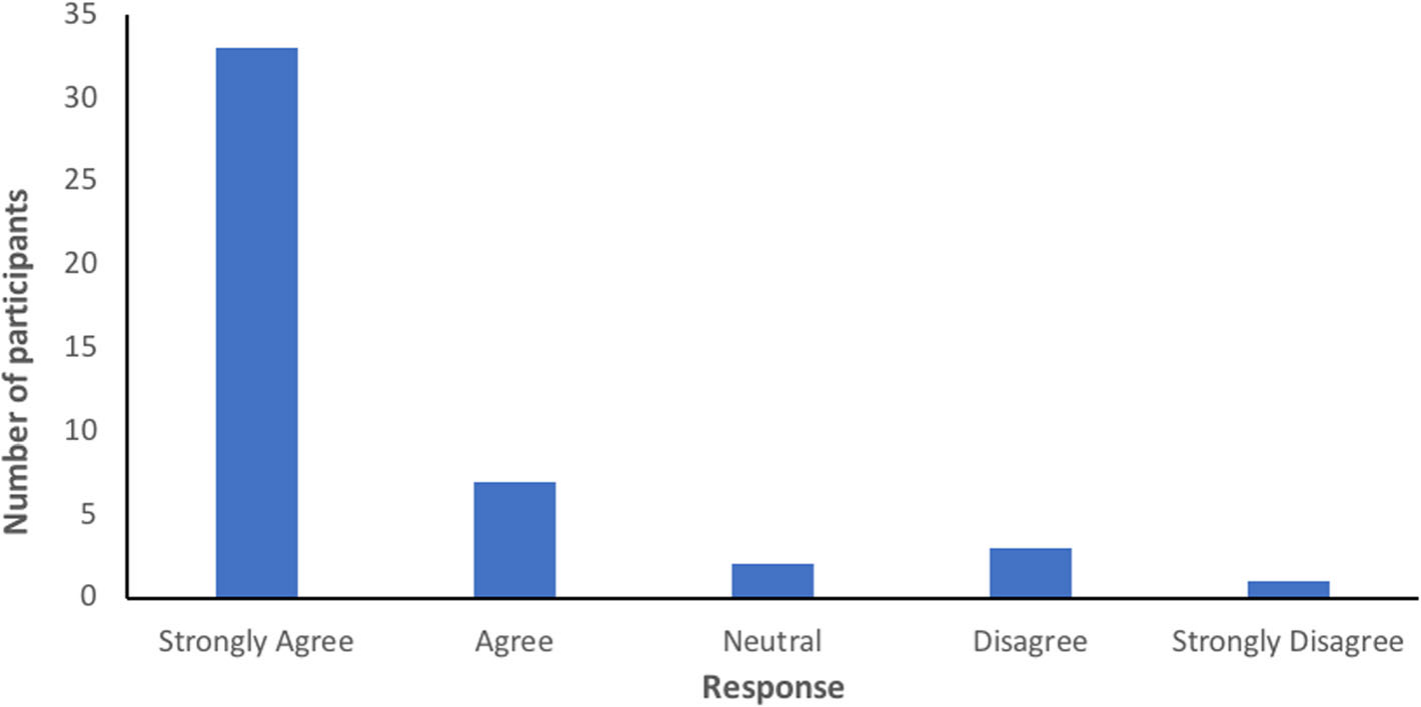
Responses to the statement, ‘The patch is comfortable to wear’

**Fig. 3:**
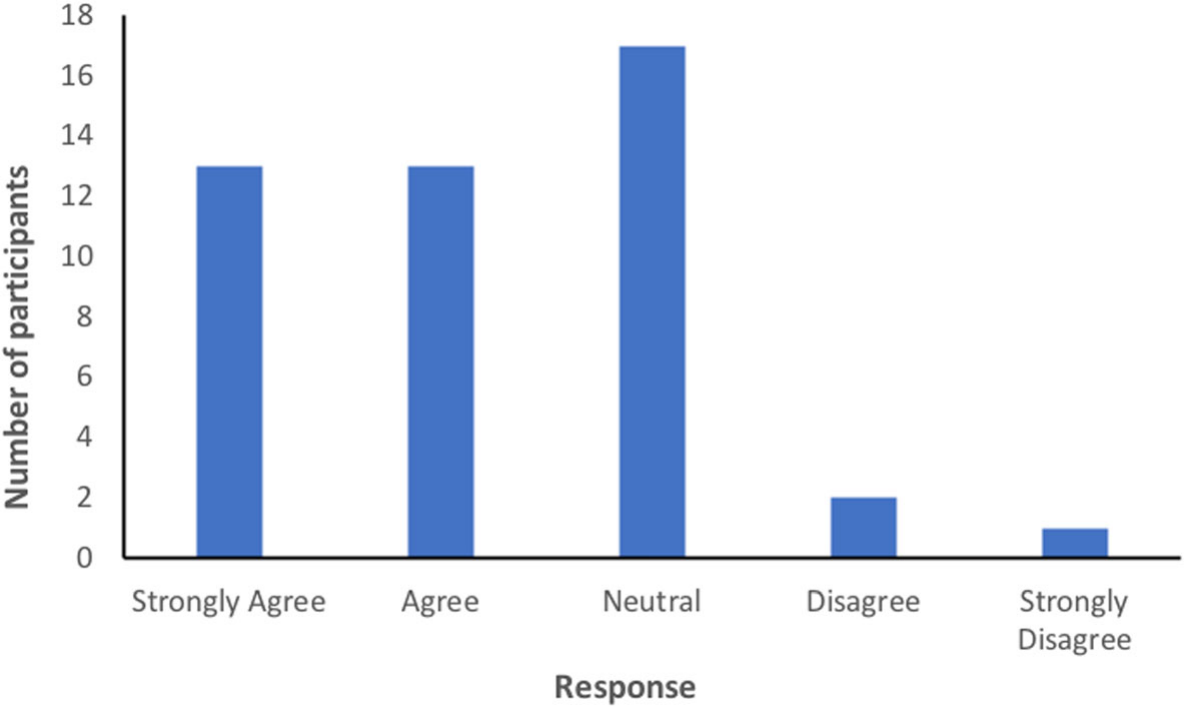
Responses to the statement, ‘I felt safer wearing the patch’

Of the 52 participants who wore the patch, 7 discontinued wear due to discomfort (*n* = 5) or inconvenience from false alerts (*n* = 2). The patient acceptability questionnaire was completed by 46 participants (88.5%). Fourteen participants did not complete the questionnaire as they were discharged from hospital before the questionnaire was administered. Most participants found the patch comfortable and felt safer wearing it, as shown in Figs. 2 and 3.

### Cost-effectiveness

**Fig. 4:**
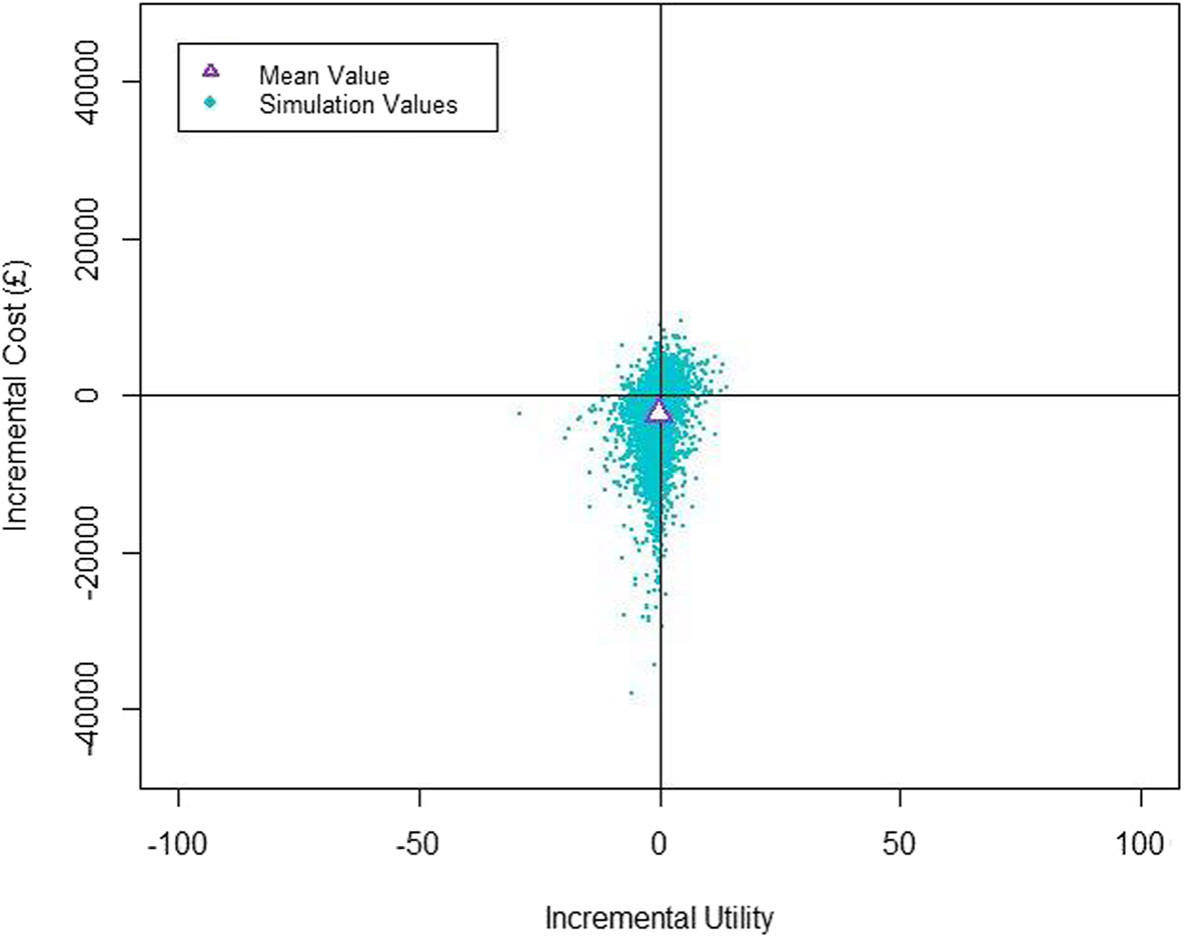
Cost-effectiveness plane

At the 6-week time horizon, the SensiumVitals® remote monitoring system was cost-saving when compared to standard NEWS monitoring from an NHS payer perspective. The ICER was £1,460 (95% CI − £6,780, £9,701) per every one-point increase in overall quality of life on the abbreviated World Health Organization Quality of Life (WHOQOL-BREF) score. For the probabilistic sensitivity analysis, the results of the Monte-Carlo simulations are shown in the reference. This analysis indicates that the probability of cost-saving is 69.9% and the probability of benefit to quality of life is 58% (Fig. 4).

### Progression criteria

The criteria for progression to a definitive randomised controlled trial were met.

- The recruitment of over 120 patients was achieved well within 12 months. The study opened to recruitment on 20^th^ October 2017 and closed on 10^th^ April 2018 with 125 participants receiving monitoring on the trial.
- Missing data was limited only to questionnaire responses; no participants were lost to follow-up. One participant was withdrawn due to loss of capacity.

## Discussion

In this single-centre randomised controlled feasibility trial, the feasibility of performing a large-scale randomised controlled trial of continuous remote monitoring after major surgery has been confirmed.

A number of uncertainties on feasibility existed prior to this study: feasibility of recruitment, estimated recruitment rate, levels of adherence to protocol, estimated amount of missing data and potential optimal outcome measures. These uncertainties have been addressed by the study’s key findings.

The recruitment target was met within 6 months with a high rate of eligibility and consent; the recruitment rate was higher than expected. The number of patients classed as ‘drop-out’ due to design were less than anticipated and no participants crossed over into the alternative trial allocation group. Missing data was limited only to questionnaire responses; no participants were lost to follow-up and one participant was withdrawn due to loss of capacity.

The rate of critical care admissions and length of hospital stay are potential primary outcome measures for a definitive trial. Length of hospital stay data are applicable to every participant and, using the summary statistics from this feasibility study, shows a high likelihood of demonstrating efficacy over intermittent monitoring alone. It is an outcome measure that is relevant for the individual patient, society and the healthcare system. However, the effect on length of hospital stay may not be directly attributable to the intervention.

In contrast, the rate of critical care admissions is an outcome measure that can more easily be attributed to the intervention. By alerting the healthcare provider at the earliest sign of deterioration, continuous monitoring may detect complications earlier than intermittent vital signs monitoring, allowing for prompt treatment and potentially reducing the need for Level II/III care. However, the event rate, even in a high-risk population, is likely to be low, necessitating an inflated sample size with its associated costs; however, recruitment to a higher target is likely to be successful given the high consent rates in the feasibility study. Practically, it may be difficult to implement given that different hospitals have different admission and discharge criteria to critical car e[14], which may produce a further clustering effect.

In this study, the intra-cluster correlation co-efficient and the balanced preoperative demographics between arms suggest that the randomisation method employed in the trial was appropriate. Of interest is the fact that, despite using ASA grade as a stratification factor, there were more postoperative complications in the control arm than the intervention arm in every Clavien-Dindo classification group. Whilst the difference in the number of participants experiencing major complications (Clavien-Dindo III or IV) could be explained by the intervention preventing escalation of care in the event of complications, the difference in the minor complications (Clavien-Dindo I and II) is less predictable. Possible causes of these differences include the intervention preventing escalation of complications; a failure of randomisation, which may indicate that the ASA score is not sufficiently specific to stratify participants in terms of their risk; or that the complications occurred by chance, in which case larger numbers of participants would be required to avoid this risk in a definitive trial.

The moderate ICC estimate indicates that there is some effect of clustering on the endpoint selected (length of hospital stay) based on which bay a participant was admitted to. The ICC and the confidence interval limits were used to calculate a sample size range for a potential definitive trial. Using mean length of hospital stay as the primary endpoint would require a sample size of 602 participants (301 per arm), which provides 80% power at the 5% level of significance to detect a 2-day difference in length of hospital stay, allowing for 15% attrition to account for participants who drop out by design and those who withdraw from monitoring during the study (12.8% of the study sample in the feasibility trial). The sample size calculation uses the ICC estimated from the feasibility study, and refers to bays as the clusters in question. In a definitive trial, clusters may be larger; for instance, randomisation may be clustered by wards rather than bays. In this case, larger clusters may require higher numbers of participants to maintain 80% power.

A secondary objective was to evaluate the safety, potential efficacy and acceptability of a wearable, remote monitoring system for patients after major surgery, as compared to standard monitoring with the NEWS system alone. Previous studies evaluating continuous monitoring of multiple vital signs parameters have shown mixed results. An industry-funded controlled before-and-after study of 7643 patients [15] found that continuous monitoring on a medical-surgical unit was associated with a decrease in total ICU days, but the rate of ICU admission was unchanged. A randomised controlled trial of 402 high-risk medical and surgical patients found that continuous multi-parameter monitoring showed no effect on adverse events or mortality [3].

In this study, participants who had undergone major abdominal surgery were less likely to have an unplanned critical care admission and had a shorter average length of hospital stay if they received continuous vital signs monitoring when compared to those receiving usual intermittent monitoring alone. This could be attributed to the earlier detection of complications preventing escalation of care to Level II/III wards and prolonging patient recovery. There was no difference in the time taken to receive antibiotics in cases of sepsis. The cost-utility analysis indicated that the SensiumVitals® remote monitoring system had the potential to be cost-saving when compared to standard NEWS monitoring alone.

Subgroup analyses were performed in order to delineate which patients would benefit most from continuous monitoring. Patients were divided into ‘high-risk’ and ‘low-risk’ categories based on their ASA score and whether they had a planned perioperative critical care admission; these two factors are known to be indicators of risk in surgical patients [16]. This subgroup analysis showed that the difference in length of hospital stay was particularly pronounced in ‘low-risk’ patients. This finding is limited by the small subgroup numbers, but could indicate that this group may be most likely to benefit from continuous remote monitoring, especially if their ‘high-risk’ counterparts already receive extra clinical attention due to their perceived risk of deterioration.

The findings must be interpreted within the limitations of the study. Due to the feasibility nature of the study, a formal sample size calculation was not required and the findings were limited to descriptive statistics; no formal statistical comparison was possible [17]. Although the nature of this study does not permit conclusions to be drawn about the efficacy of the intervention, the observations give sufficient confidence that further evaluation within a larger randomisation comparison is justified. Such a study should consider preoperative risk factors for complications, in addition to ASA grade, as stratification factors to ensure that groups are balanced in terms of frequency of complications. Consideration should also be given to maximising protocol adherence in the intervention group.

The potential benefits of continuous monitoring may have been underestimated in this study due to the exposure to the patch in the intervention arm. Although most patients found the patch comfortable and felt safer wearing it, eight participants withdrew from the intervention before monitoring had commenced and a further nine participants discontinued the intervention before discharge and within 5 days of application. Adherence to the monitoring protocol could be improved in a future trial by optimising patient comfort through choice of device, minimising the number of false alerts through better signal processing and encouraging participation until discharge through regular bedside visits.

The cost-utility analysis was limited by the amount of data concerning the influence of post-operative complications on quality of life. The results are based on the findings of a single study which used the WHOQOL-BREF instrument to measure quality of life following colorectal surgery [18]. It was not possible to calculate quality of life in terms of quality-adjusted life years (QALYs), and therefore it was inappropriate to apply a willingness-to-pay threshold to the cost-effectiveness plane. As a consequence, the ICER has limited usefulness and there is considerable uncertainty in the model, reflected by the large confidence interval surrounding the ICER. Future evaluations should include quality of life measurements using the EuroQol five dimension scale (EQ-5D). The National Institute for Clinical Excellence (NICE) Guide to the Methods of Technology Appraisal expresses a preference for using the EQ-5D for adult populations to estimate the QALY impact of different technologies [19].

In conclusion, this study has demonstrated the feasibility of performing a large-scale randomised controlled trial of continuous remote monitoring after major surgery. The purpose of this study was not to assess the clinical efficacy of the intervention, but the observed differences in the length of stay between the two groups suggest that this might serve as an appropriate end-point for a larger study. This must be balanced against the small number of participants and the potential failure of randomisation in the study.

The findings of this feasibility study suggest that progression to a definitive multicentre randomised controlled trial would be appropriate, with reassuringly high rates of patient recruitment. Participants should be individually randomised and stratified to minimise the baseline differences between the two treatment arms; ASA might be replaced by more specific risk stratification tools such as the PPOSSUM (Physiological and Operative Severity Score for the enumeration of Mortality and morbidity) score [20]. The ICC should be taken into account in the sample size calculation to account for potential clustering of outcomes at bay or ward level. Care should also be taken to monitor and address inadequacies in other areas that might mask the potential benefit of additional monitoring, such as patient adherence.

## Supplementary Information

**Additional file 1:.** Decision tree for cost-utility analysis.

**Additional file 2:.** Details of surgical procedures received by participants in the TRaCINg study.

**Additional file 3:.** Details of surgical procedures received by participants in the TRaCINg study.

**Additional file 4:.** Sources of confirmed sepsis (some participants experienced more than one source per sepsis event).

## Data Availability

The datasets used and/or analysed during the current study are available from the corresponding author on reasonable request.

## Abbreviations

ASA: American Society of Anaesthesiologists (score for pre-operative functional status)
CTRU: Clinical Trials Research Unit
EQ-5D: EuroQol five dimension scale
HDU: High dependency unit
ICER: Incremental cost-effectiveness ratio
ICU: Intensive care unit
ITT: Intention-to-treat
mITT: Modified intention-to-treat
NEWS: National Early Warning Score
NICE: The National Institute for Clinical Excellence
PSA: Probabilistic sensitivity analysis
QALY: Quality-adjusted life year
RCT: Randomised controlled trial
WHOQOL-BREF: World Health Organization Quality of Life (abbreviated)
