# Supplementary figures and images for: Trial of remote continuous versus intermittent NEWS monitoring after major surgery (TRaCINg): a feasibility randomised controlled trial
Source: Pilot Feasibility Stud. 2020 Nov 23;6:183. doi: 10.1186/s40814-020-00709-8 (PMC7684886; doi:10.1186/s40814-020-00709-8)

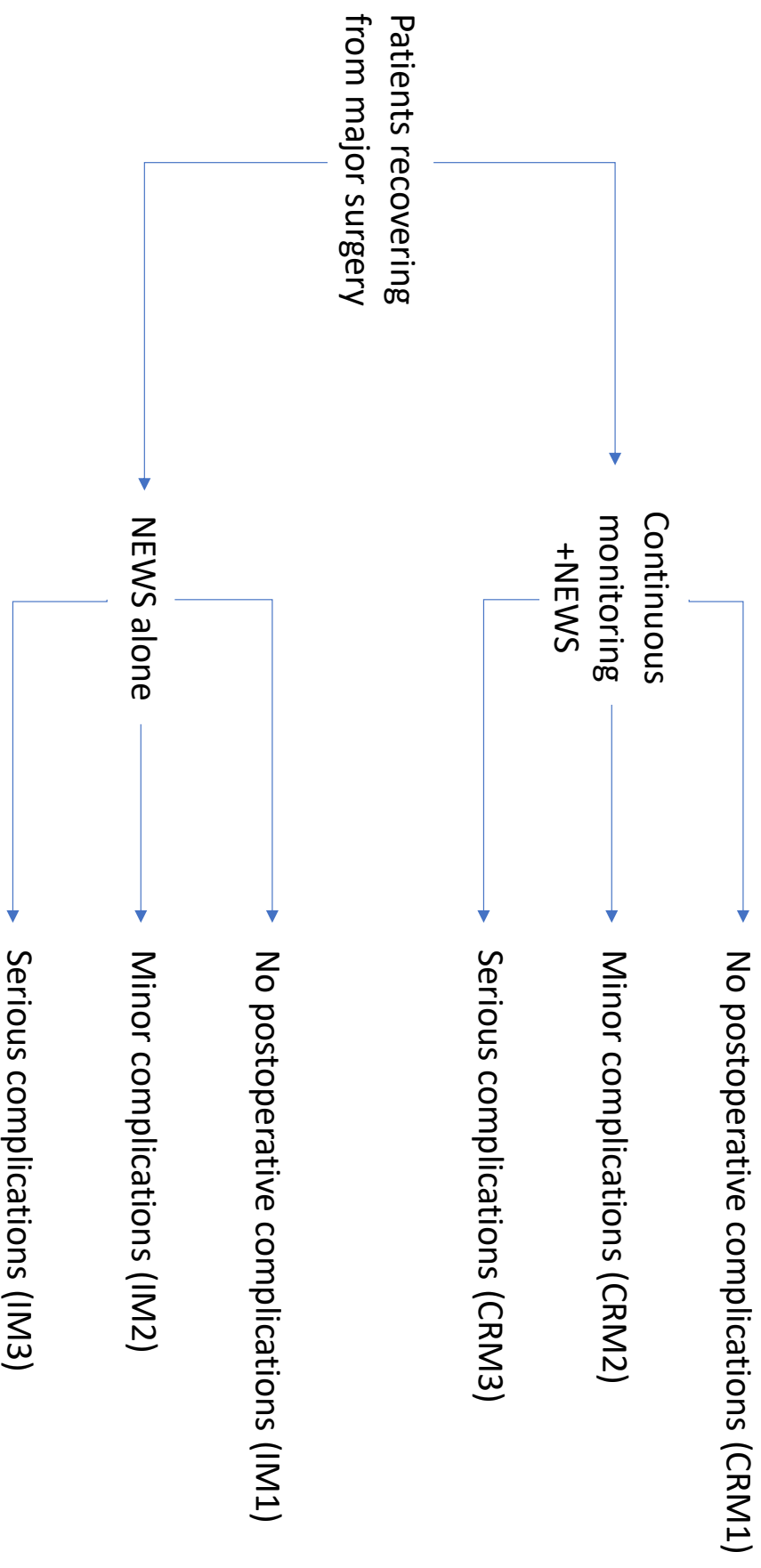

Supplement: Supplementary file 1 — Additional file 1:. Decision tree for cost-utility analysis. [file 40814_2020_709_MOESM1_ESM.pdf]
