# Supplementary material for: Trial of remote continuous versus intermittent NEWS monitoring after major surgery (TRaCINg): a feasibility randomised controlled trial
Source: Pilot Feasibility Stud. 2020 Nov 23;6:183. doi: 10.1186/s40814-020-00709-8 (PMC7684886; doi:10.1186/s40814-020-00709-8)
Supplement: Supplementary file 2 — Additional file 2:. Details of surgical procedures received by participants in the TRaCINg study. [file 40814_2020_709_MOESM2_ESM.docx]

Details of surgical procedures received by participants in the TRaCINg study
