# Supplementary material for: Trial of remote continuous versus intermittent NEWS monitoring after major surgery (TRaCINg): a feasibility randomised controlled trial
Source: Pilot Feasibility Stud. 2020 Nov 23;6:183. doi: 10.1186/s40814-020-00709-8 (PMC7684886; doi:10.1186/s40814-020-00709-8)
Supplement: Supplementary file 3 — Additional file 3:. Details of surgical procedures received by participants in the TRaCINg study. [file 40814_2020_709_MOESM3_ESM.docx]

**Sample size calculations**

To provide 80% power, the sample size could range from 84 participants per arm (using the lower limit of the 95% confidence interval for the ICC and the upper limit of the 95% confidence interval for the chosen endpoint) to 3719 participants per arm (using the upper limit of the 95% confidence interval for the ICC and the lower limit of the 95% confidence interval for the chosen endpoint)(see Table 6).

To provide 90% power, the sample size could range from 111 participants per arm (using the lower limit of the 95% confidence interval for the ICC and the upper limit of the 95% confidence interval for the chosen endpoint) to 4978 participants per arm (using the upper limit of the 95% confidence interval for the ICC and the lower limits of the 95% confidence intervals for the chosen endpoint)(see Table 7).

|  | | Design effect (DE) | | |
| --- | --- | --- | --- | --- |
|  |  | Using lower 95% CI limit of ICC (*ICC_low_* =0, *DE_low_* =1) | Using ICC  (*ICC*=0.06, *DE* = 1.36) | Using upper 95% CI limit of ICC (*ICC_upp_* =0.2, *DE_upp_* = 2.14) |
| Sample size calculation using length of hospital stay as primary endpoint | Lower 95% CI limits  (*LCI_C_* =11.3, *LCI_T_* =9.5) | 1738 | 2364 | 3719 |
|  | Mean  (*M_C_* =16.2, M_T_=11.6) | 222 | 301 | 474 |
|  | Upper 95% CI limits  (*UCI_C_* =21.2, *UCI_T_* =13.7) | 84 | 114 | 179 |

Table 2: Participants required per group to provide 80% power. ICC_low_ = lower limit of ICC, ICC = point estimate of ICC, ICC_upp_ = upper limit of ICC, DE_low_ = lower limit of DE, DE= point estimate of DE, DE_upp_ = upper limit of DE, LCI_C_ = control group lower limit, LCI_T_ = intervention group lower limit, M_C_= control group mean, M_T_= intervention group mean, UCI_C_ = control group upper limit, UCI_T_ = intervention group upper limit.

|  | | Design effect (DE) | | |
| --- | --- | --- | --- | --- |
|  |  | Using lower 95% CI limit of ICC (*ICC_low_* =0, *DE_low_* =1) | Using ICC  (*ICC*=0.06, *DE* = 1.36) | Using upper 95% CI limit of ICC (*ICC_upp_* =0.2, *DE_upp_* = 2.14) |
| Sample size calculation using length of hospital stay as primary endpoint | Lower 95% CI limits  (*LCI_C_* =11.3, *LCI_T_* =9.5) | 2326 | 3164 | 4978 |
|  | Mean  (*M_C_* =16.2, M_T_=11.6) | 297 | 404 | 635 |
|  | Upper 95% CI limits  (*UCI_C_* =21.2, *UCI_T_* =13.7) | 111 | 151 | 237 |

Table 3: Participants required per group to provide 90% power. ICC_low_ = lower limit of ICC, ICC = point estimate of ICC, ICC_upp_ = upper limit of ICC, DE_low_ = lower limit of DE, DE= point estimate of DE, DE_upp_ = upper limit of DE, LCI_C_ = control group lower limit, LCI_T_ = intervention group lower limit, = control group mean, = intervention group mean, UCI_C_ = control group upper limit, UCI_T_ = intervention group upper limit.
