# Supplementary material for: Trial of remote continuous versus intermittent NEWS monitoring after major surgery (TRaCINg): a feasibility randomised controlled trial
Source: Pilot Feasibility Stud. 2020 Nov 23;6:183. doi: 10.1186/s40814-020-00709-8 (PMC7684886; doi:10.1186/s40814-020-00709-8)
Supplement: Supplementary file 4 — Additional file 4:. Sources of confirmed sepsis (some participants experienced more than one source per sepsis event). [file 40814_2020_709_MOESM4_ESM.docx]

| **Source of sepsis** | **NEWS alone**  **n=65** | **SensiumVitals^®^** **+ NEWS**  **n=60** | **Total**  **n=125** |
| --- | --- | --- | --- |
| *UTI* | 0 | 3 | 3 |
| *Anastomotic leak* | 2 | 0 | 2 |
| *Pneumonia* | 5 | 5 | 10 |
| *Wound* | 2 | 1 | 3 |
| *Collection* | 4 | 2 | 6 |

Table 4: Sources of confirmed sepsis (some participants experienced more than one source per sepsis event)
